# Imaging the Premonitory Phase of Migraine

**DOI:** 10.3389/fneur.2020.00140

**Published:** 2020-03-25

**Authors:** Nazia Karsan, Peter J. Goadsby

**Affiliations:** ^1^Headache Group, Department of Basic and Clinical Neuroscience, Institute of Psychiatry, Psychology and Neuroscience, King's College London, London, United Kingdom; ^2^NIHR-Wellcome Trust King's Clinical Research Facility and SLaM Biomedical Research Centre, King's College London, London, United Kingdom

**Keywords:** migraine, premonitory, neuroimaging, headache, prodrome

## Abstract

Migraine is a common and disabling brain disorder with a broad and heterogeneous phenotype, involving both pain and painless symptoms. Over recent years, more clinical and research attention has been focused toward the premonitory phase of the migraine attack, which can start up to days before the onset of head pain. This early phase can involve symptomatology, such as cognitive and mood change, yawning, thirst and urinary frequency and sensory sensitivities, such as photophobia and phonophobia. In some patients, these symptoms can warn of an impending headache and therefore offer novel neurobiological insights and therapeutic potential. As well as characterization of the phenotype of this phase, recent studies have attempted to image this early phase using functional neuroimaging and tried to understand how the symptoms are mediated, how a migraine attack may be initiated, and how nociception may follow thereafter. This review will summarize the recent and evolving findings in this field and hypothesize a mechanism of subcortical and diencephalic brain activation during the start of the attack, including that of basal ganglia, hypothalamus, and thalamus prior to headache, which causes a top-down effect on brainstem structures involved in trigeminovascular nociception, leading ultimately to headache.

## Introduction

Migraine is a disabling condition that, in addition to headache, also involves often disabling non-headache symptoms. Although the pain phase of the migraine attack is well-recognized and characterized by moderate to severe headache with associated sensory sensitivities such as photophobia and phonophobia, and nausea and vomiting (1), increasingly, associated symptomatology such as cognitive dysfunction (2) and fatigue (3) are recognized to contribute to attack-related disability. It has been recognized for over a century that non-painful symptomatology can precede the migraine attack (4), but only over recent decades has the phenotype and prevalence of early attack symptoms been captured in both adults and children in detail (5–18), and the ability to reliably and reproducibly predict headache onset has been explored (8, 9, 19). This early phase of the migraine attack, when symptomatology outside of pain manifests, provides novel neurobiological and therapeutic insights into possible mechanisms behind attack initiation in a genetically predisposed individual, and into treatments that may work at aborting or preventing pain before its onset. Given migraine therapeutics is an evolving field, fundamental understanding of the underlying neurobiological mechanisms behind migraine attack initiation is key to advancing abortive therapeutics further by developing migraine-specific agents that are likely to be more efficacious and tolerable than currently available options.

Neurophysiological studies have suggested that the brain is already electrically different in the lead-up to migraine pain (20–24). One of the other ways in which the premonitory phase of the migraine attack has been studied has been with the increasing use of functional neuroimaging. This evolving field has allowed understanding of human disease in patients and has been pivotal in furthering understanding of several disorders. The functional neuroimaging “signature” of the pain phase of the migraine attack has been reproduced across several studies since the 1990's (25–29). Over the last few years, various methodologies have also been used in imaging the premonitory phase of both spontaneous and exogenously triggered migraine attacks and have alluded to early involvement of subcortical diencephalic and brainstem structures and their role in attack initiation (30).

This review will summarize these studies and hypothesize a possible network of brain dysfunction, which starts prior to pain onset and continues throughout migraine headache, and most likely even following headache resolution until the brain reverts to its true interictal state and the patient symptomatically feels back to normal function.

## Perfusion Imaging

**Figure 1:**
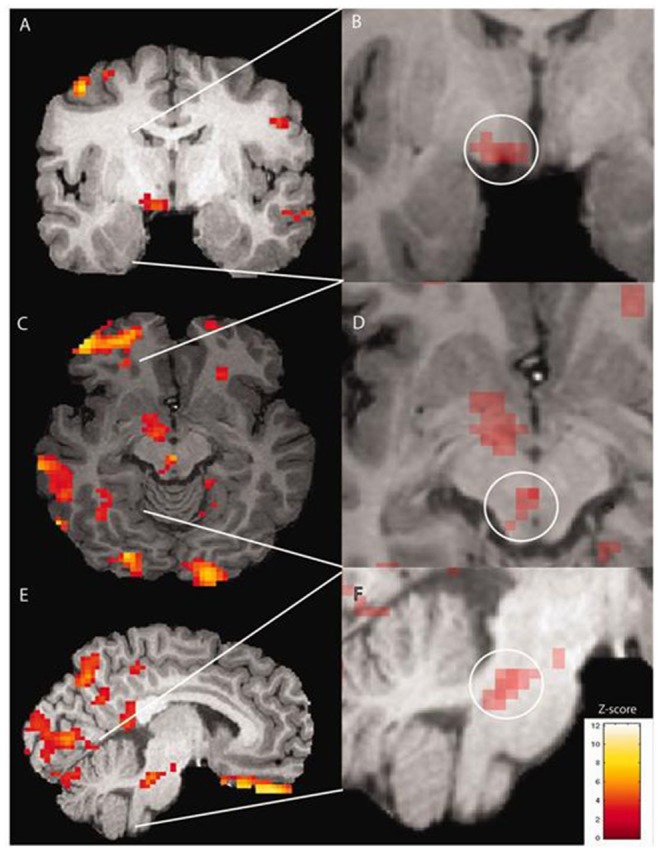
Brain activations on PET imaging during the early premonitory phase of nitroglycerin-triggered migraine attacks, taken from Maniyar et al. (31) study.

Various methods of functional neuroimaging, that is, using neuroimaging methodologies to understand human brain function, exist. Perfusion is one measure used as a surrogate for neuronal activity, on the basis that the more a region of brain is active, the more blood supply that area will require and the higher will be the regional blood flow in that area, which can be mapped with perfusion imaging using various modalities. One such modality is positron emission topography (PET), which can be used to assess perfusion if used along with a radioisotope of labeled water (${\text{H}}_{2}^{15}$O). The first PET study using blood flow measurement in the premonitory phase of migraine was conducted in 2014 by Maniyar et al. (31). The authors used this imaging methodology to study the premonitory phase of nitroglycerin-triggered migraine in eight subjects in a repeated measures design. Despite the lack of a healthy control arm, the study was able to identify statistically significant areas of increased blood flow during both the early and late premonitory symptoms in the study subjects, in brain areas including the hypothalamus, thalamus, cingulate cortex, and dorsolateral pons, all areas that are believed to be important in the pain phase of migraine from other studies (25–27, 32). For the first time, this study provided a central and neuronal surrogate correlate for what patients experience during the premonitory phase [see Figure 1, reused with journal copyright permission, taken from Maniyar et al. (31)].

The same authors completed additional studies looking at some symptoms in particular, namely, nausea and photophobia. Using similar methodologies, the authors compared subjects with and without each symptom during the premonitory phase to try and elucidate which part of the brain these individual symptoms could be arising from (33, 34). The authors were able to hypothesize that photophobia, or specifically photic hypersensitivity (that is, a sensitivity or aversion to light without light exacerbating pain), involved the occipital lobes, which displayed increased perfusion in subjects with the symptom relative to those without (33). Similarly, nausea involved activations in a region including the nucleus tractus solitarius in the medulla, an area known as the chemoreceptor trigger zone. This area showed increased blood flow in those with nausea during the premonitory phase relative to those without (34).

A more recent study utilized perfusion MRI, using arterial spin labeling (ASL), to evaluate brain blood flow changes during the premonitory phase (35). This study again used nitroglycerin-triggered migraine attacks, because of the logistical issues with repeatedly and reliably capturing spontaneous premonitory symptoms. The authors studied premonitory symptoms in 25 subjects and compared the premonitory scans following nitroglycerin with scans obtained at the same time following placebo in 21 subjects to correct for any possible nitroglycerin-induced changes on cerebral blood flow. Similar areas of increased blood flow (and therefore the suggestion of increased neuronal activity) were identified as the Maniyar et al. study (31), including the hypothalamus, thalamus, basal ganglia, and limbic cortex, during the premonitory phase, supporting the theory of subcortical brain dysfunction in migraine attack initiation.

## Functional MRI

The majority of the imaging work done in this field has used functional MRI approaches—either with external stimulation or without. The very first study was conducted by Stankewitz et al. and did not aim to study premonitory symptoms specifically (36). The study used trigemino-nociceptive stimulation with intranasal gaseous ammonia in migraineurs in the interictal state, and also in the preictal (12–48 h prior to the next migraine attack) and ictal states in *post-hoc* analyses, thereby capturing the lead-up to the headache phase of migraine. The study showed increased neuronal activity, measured using the blood oxygen level-dependent (BOLD) contrast signal intensity, in the region of the spinal trigeminal nucleus in the preictal phase in migraineurs relative to the interictal phase and during the acute pain phase. Rostral pontine activation was only seen during acute pain, and not outside of pain. Interestingly, the intensity of the BOLD signal in response to nociceptive stimulation was able to predict the next headache, in that the stronger the BOLD response in the region of the spinal trigeminal nuclei, the closer the next headache would be.

**Figure 2:**
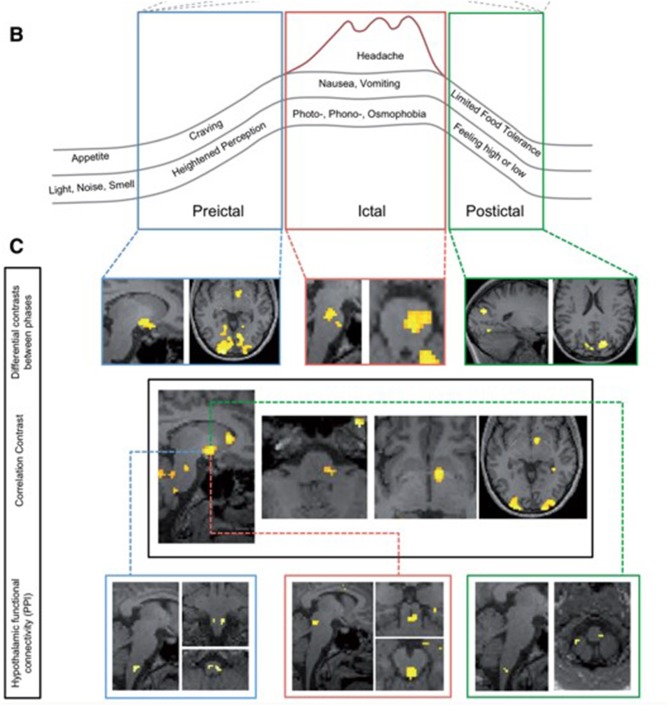
Changes during the migraine cycle, taken from the Schulte et al. study (37). **(A)** Unpleasantness ratings for ammonia, rose odor, and checkerboard stimulation (red line and dots: ammonia-ratings; green line and crosses: rose odor ratings; blue line and asterisks: checkerboard ratings), with higher values representing a more unpleasant experience. Red areas: days of migraine pain with varying red color intensities indicating different intensities of migraine pain and blue areas representing the last scan before onset of migraine pain. **(B)** Overview of the migraine cycle. **(C)** Results from functional MRI.

Other studies have followed, studying the preictal or pre-headache phase of the migraine attack using fMRI approaches. An impressive study conducted by Schulte and colleagues in 2016 aimed to study further the theory of possible oscillatory brainstem responses during the migraine cycle and studied the same individual with daily scanning for 30 days, thereby capturing three spontaneous migraine attacks and the periods surrounding these (37). This study demonstrated an increase in hypothalamic activity in the period prior to headache in response to the same intranasal gaseous ammonia nociceptive stimulation. There was also altered functional connectivity between the hypothalamus and other migraine brain areas, including the spinal trigeminal nuclei region and the dorsal rostral pons during the day preceding headache. This study provided additional information to the prior study and suggested that as well as altered brainstem responses, altered hypothalamic and brainstem connectivity could be involved in the start of a migraine attack. Again, although this study did not phenotype the subject extensively before the pain-free scanning days, the lead-up phase to a migraine headache was captured and assessed using imaging. See Figure 2, reused with journal permission, taken from Schulte and May (37). The authors have recently presented an extension to this work by replicating the study in a further eight subjects who have also been imaged daily for 30 days, with 15 spontaneous migraine attacks captured, and have found additional results for increased functional connectivity between the right nucleus accumbens and left amygdala, hippocampus, and parahippocampal gyrus preictally compared with interictally, as well as increased connectivity between the right nucleus accumbens and dorsal rostral pons (38). These findings support theories of dopamine pathway involvement in the premonitory phase, as well as the involvement of the hypothalamus and limbic pathways.

Another study followed in 2018 and was conducted by Meylakh et al. (39). The authors used various fMRI approaches (infraslow oscillatory activity, connectivity, and regional homogeneity) to study spontaneous migraine preictally and postictally, as well as interictally and in healthy controls. The authors were able to demonstrate increased infraslow oscillatory activity in the brainstem and hypothalamic regions just before migraine headache, including the spinal trigeminal nucleus, dorsal pons, and hypothalamus. There was also increased functional coupling between these areas just before pain, as well as increased regional homogeneity. This finding was not present interictally, postictally, or in healthy controls. This study provided supportive evidence for oscillatory brainstem and hypothalamic activity in the lead-up to migraine headache and proposed a potential role for astrocytic involvement in attack initiation, rather than neuronal. Similarly, to previous studies, this study did not assess functional correlation, as subjects were not phenotyped in detail as to what, if anything, they were experiencing symptomatically on the preictal scan days. However, similar regions of interest emerge for the premonitory or pre-headache lead-up to the migraine as previous studies, and indeed as for previous migraine headache imaging studies.

A recent study performed fMRI using orofacial nociceptive stimulation in 31 migraine patients and 31 healthy controls during different parts of the migraine attack (40). There was an increase in pain ratings in response to nociceptive stimulation in the lead-up to the next migraine attack, and then these ratings decreased immediately prior to pain. Imaging responses in the spinal trigeminal nuclei dramatically increased in the 24 h prior to pain onset in response to noxious stimulation, with reduced functional connectivity between this region and the rostral ventral medulla. This study therefore suggests a pre-headache sensitivity to pain, or reduced threshold to pain, during the migraine attack (and therefore a susceptibility to exogenous triggers) and the subsequent implication of altered or dysfunctional endogenous pain modulation within the pain network in the brain during the migraine attack or cycle, which may be responsible for the sensation of pain felt following premonitory symptoms. The same authors have also recently demonstrated possible structural changes in similar brain areas (dorsolateral pons, periaqueductal gray, and spinal trigeminal nuclei) within 24 h of a migraine attack using mean diffusivity and fractional anisotropy (41).

We have recently presented our resting-state fMRI results looking at seed-based functional connectivity with the BOLD contrast, in nitroglycerin-triggered premonitory symptoms relative to placebo, and shown increased thalamocortical connectivity and functional uncoupling between the pons and the limbic lobe in the premonitory phase, with increased functional coupling between the pons and spinal trigeminal nuclei during migraine headache, thus suggesting changing alterations in subcortical and brainstem networks in the premonitory phase (42).

The imaging studies of the premonitory phase are summarized in Table 1.

**Table 1:** Comparison of the imaging findings of the different studies examining the premonitory or lead-up to headache phase of the migraine attack.

**Brain region**	**Stankewitz et al. (36)** **Task-evoked fMRI (trigemino-nociceptive stimulation)**	**Schulte et al. (37)** **Task-evoked fMRI (trigemino-nociceptive stimulation)**	**Schulte et al. (38)** **Task-evoked fMRI (trigemino-nociceptive stimulation)**	**Maniyar et al. (31)** **${\text{H}}_{2}^{15}$O perfusion PET (observed triggered attacks)**	**Karsan et al. (35)** **Perfusion arterial spin-labeled MRI (observed triggered attacks)**	**Meylakh et al. (39)** **Resting-state fMRI (infraslow oscillatory activity, regional homogeneity, and connectivity)**	**Marciszewski et al. (40)** **Task-evoked brainstem responses (noxious orofacial stimulation) and resting-state fMRI connectivity**	**Marciszewski et al. (41)** **Diffusion tensor imaging (DTI) and fractional anisotropy (FA)**	**Karsan et al. (42)** **Resting-state BOLD-fMRI (observed triggered attacks)**
**Hypothalamus**		**+**	**+**	**+**	**+**	**+**			
Thalamus				+	+			+	+
**Ventral tegmentum**		**+**		**+**	**+**	**+**		**+**	
Caudate				+	+				
Putamen				+	+				
Pallidum				+	+				
Nucleus accumbens			+		+				
**Spinal trigeminal nucleus**	**+**	**+**				**+**	**+**	**+**	
Medulla								+	+
**Dorsal pons**		**+**	**+**	**+**		**+**		**+**	**+**
Frontal cortex				+	+				+
Precuneus/cuneus				+					+
Cerebellum				+					
Anterior cingulate				+	+				+
Occipital cortex				+	+				
Temporal cortex, including amygdala and hippocampus			+	+	+				

*The brain regions that have been suggested to be implicated in five or more studies have been highlighted in bold text*.

## Functional and Neurobiological Correlation With the Symptomatic Premonitory Phase

Although only few of these studies, namely, the perfusion studies, have phenotyped patients with regard to premonitory symptomatology prior to scanning, the similarities between implicated brain areas between all the studies and methodologies are striking and supports a network of altered brain activity involving pain processing, sensory integration, and limbic areas prior to migraine pain. These regions are likely functionally correlated to the symptomatology patients report during this phase. It can be reasonably assumed that mood and cognitive change may come from limbic pathway involvement, yawning and sleep disturbance may be mediated via the hypothalamus, and photophobia, allodynia, and other sensory sensitivities may arise from thalamocortical connections (30, 43–47).

## Premonitory Symptoms, Attack Initiation, and Nociception

Although the ictus of the migraine attack is usually thought of as the pain phase, the onset of the attack could be regarded as when the brain is different to its interictal state, and this is likely to be days before the onset of pain, as suggested by neurophysiological studies. How the changes in brain function occur, therefore, are the answer to how an attack is mediated; and perhaps how these changes go from producing premonitory symptoms to producing pain is a second question. Many of the symptoms that patients report during the premonitory phase, including fatigue, cognitive dysfunction, and even photophobia, can also be present interictally in some individuals; and in our nitroglycerin-triggered experimental work, we have demonstrated the ability of the drug to provoke premonitory-like symptoms with no delayed migraine headache thereafter (48). This alludes to a possible intra-attack threshold, which could be explained by the oscillatory networks of brain activity demonstrated through the imaging studies discussed in this review, and suggests that as the networks of brain activity passing through the hypothalamus, brainstem, and cortex change throughout the migraine cycle, so do the pain thresholds. Although agents such as nitroglycerin and pituitary adenylate cyclase activating peptide (PACAP) can therefore trigger premonitory symptoms (49, 50), perhaps the likelihood of headache following thereafter is based on endogenous pain modulation systems and an individual's genetic and environmental susceptibility to developing a migraine headache that day.

## Premonitory Symptoms and Aura

Although in some patients aura can be a warning of an impending headache, it is clear that aura can occur anytime during the migraine attack and indeed in the absence of headache too (51). Clinically, it is feasible that aura and premonitory symptoms may seem to overlap in patients who experience aura prior to headache. Aura is defined as the presence of gradually developing focal and transient neurological symptoms (1), and these can be positive or negative phenomena, whereas premonitory symptoms suggest a more global disturbance of brain function, without clear lateralizing neurological deficit, and with symptoms in general lasting longer than aura and likely occurring in a higher proportion of subjects than true migraine aura. In addition, the current understanding of migraine aura is that of a cortical phenomenon, characterized by cortical spreading depression of neuronal activity, which most commonly occurs in the visual cortex (52), whilst premonitory symptoms usually coexist with each other (53) and, as discussed in this manuscript, involve more global cortical and subcortical brain dysfunction.

## Conclusions

This review has summarized the neuroimaging literature to date in the premonitory phase, or period leading up to pain, in migraine. The studies have consistently provided evidence for early brainstem involvement, as well as alluded to oscillatory brainstem, hypothalamic, and limbic networks prior to the onset of pain, with alterations in functional coupling between these regions and between pain modulatory regions such as the rostral ventral medulla and periaqueductal gray. Although the brain areas between pre-headache and during headache are similar, it is clear that thresholds may have a part to play in whether pain is perceived or not, despite similar neuroimaging findings in both the premonitory and headache phases of the attack. There is good functional correlation between the brain areas involved in the premonitory phase and the clinical phenotype of what sufferers experience during the premonitory phase of migraine.

## Author Contributions

NK collated and reviewed the existing literature and was responsible for writing the article. PG reviewed the article and provided expert opinion prior to submission. NK and PG were involved in some of the experimental work discussed in the article.

### Conflict of Interest

The authors declare that the research was conducted in the absence of any commercial or financial relationships that could be construed as a potential conflict of interest.
